# Dental Prosthetic Status and Prosthetic Need of the Institutionalized Elderly Living in Geriatric Homes in Mangalore: A Pilot Study

**DOI:** 10.5402/2011/987126

**Published:** 2010-10-11

**Authors:** Rekha P. Shenoy, Vijaya Hegde

**Affiliations:** ^1^Department of Community Dentistry, Yenepoya Dental College, Deralakatte, Mangalore 575018, Karnataka, India; ^2^Department of Community Dentistry, AJ Institute of Dental Sciences, Kuntikana, Mangalore 575004, Karnataka, India

## Abstract

*Introduction*. To promote oral health among the elderly, we need to know their prosthetic status and prosthetic need. Hence, a survey of prosthetic status and need of elderly inmates of old age homes in Mangalore was done. *Materials and Methods*. A cross-sectional study was undertaken, and 133 subjects aged 60 years and above were examined (54.9% males and 45.1% females). *Results*. Eighty-eight percent of those examined were fully edentulous, and only 12% had complete dentures; none of the study subjects had partial dentures. Prosthetic status was significantly associated with gender (*P* = .024), while prosthetic need and gender were not significantly associated (*P* = .395). *Conclusions*. A high unmet need for prosthetic care existed among the institutionalized elderly surveyed.

## 1. Introduction

 Aging is a universal process and a normal biological phenomenon. With the discoveries in the field of medical science and the improvement of social conditions, the lifespan of an individual has increased.

 Oral health can be considered as an indicator of general health and quality of life in geriatric patients. Oral diseases are progressive and cumulative. They become more complex over time. Improved oral health will allow geriatric persons to improve their self-confidence, have active social contacts, and restore the ability to work at home or at the job location. 

 It is predicted that the elderly population of India shall be the highest in the world by 2025. Their contribution to the demographic profile is increasing every day. According to the 1991 census, the geriatric population constituted 6.3% of the total Indian population [1].

 Among the many diseases and disabilities that the elderly suffer from, diseases related to the oral cavity occupy an important place. In this regard, loss of teeth in the elderly is a major concern. The problems found in them are due to a lack of treatment facilities and information on morbidity profile. In order to promote the oral health of the elderly, we need to know their prosthetic status and prosthetic need. Hence, an effort was made to collect baseline information to formulate policy and to plan, monitor, and evaluate oral health services.

## 2. Materials and Methods

This cross-sectional survey was conducted in November-December 2009 to determine the prosthetic status and prosthetic need among the institutionalized elderly residing as inmates of old age homes in Mangalore City, Karnataka State, India. All inmates aged 60 years and above formed the study population as, in India, this is the criterion for the classification of the elderly. 

 Approval for the study was obtained from the Institutional Review Board. Before conducting the survey, the investigators visited the institutions to meet the authorities in charge of these centres. Of the four old-age homes in the city, the authorities of two homes consented to the study. The aim of the study was explained, their approval was sought and obtained, and the dates for the survey were fixed. Subjects had been informed of the nature of the investigation and had been included in the study after their consent was obtained. 

 A pretested proforma was used for data collection. It consisted of two parts—the 1st part recorded data on sociodemographic factors (age and gender), while the 2nd part contained a section of the World Health Organization (WHO) Oral Health Assessment Form (1997) [2] to record the prosthetic status and prosthetic need (i.e., denture wearing and need for dentures) of the elderly population. 

 The investigators were trained and calibrated in recording the proforma and the clinical findings at Yenepoya Dental College. Standardization and calibration of the recorders was also achieved during the same sessions. The armamentarium used included mouth mirrors, instrument carrying trays, mouth masks, disposable surgical gloves, copies of the proforma, and literature for distribution. Most of the subjects were examined sitting in chairs under natural illumination; the nonambulatory were examined in their beds. Artificial illumination (torch light) was used where required.

 After completion of data collection, oral hygiene instructions were given to subjects who were capable of self-care. Oral hygiene aids and oral health education literature were handed over to the caretakers in the institutions for circulation among the inmates.

### 2.1. Statistical Analysis

Data obtained were subjected to the Statistical Package for the Social Sciences (SPSS) Version 17.0. Differences in proportions were compared using the Chi-squared test. A difference was considered to be of statistical significance if the *P*value was <.05.

## 3. Results

 A total of 133 subjects (60 female (45.1%) and 73 male (54.9%)) aged 60 years and above (mean age 71.2 ± 7.7 years) formed the study population. Those below 60 years of age (*n* = 28) were excluded from the analysis. 

 The distribution of subjects according to age and gender is given in Table 1.  Table 2 shows the distribution of study subjects according to gender and the prosthetic status of their upper and lower arches. Eighty-eight percent of the subjects had no prostheses in their upper and lower arches. Evaluation of subjects showed significant differences between the genders and the prosthetic status of their upper arches (*X* ^2^ = 5.105; *P* = .024) and lower arches (*X* ^2^ = 5.105; *P* = .024).

Table 3 shows the distribution of study subjects according to gender and the prosthetic need of their upper and lower arches. The need was highest for a full prosthesis (46.6% in the upper arch and 41.4% in the lower arch) followed by the need for a multiunit prosthesis (23.3% in upper arch and 27.1% in lower arch); 20.3% subjects did not require any prosthesis. Evaluation of subjects according to gender and the prosthetic need of their upper arches showed insignificant differences between the genders (*X* ^2^ = 8.415; *P* = .135). Again, when the study subjects were evaluated according to gender and the prosthetic need of their lower arch, the differences between the genders were insignificant (*X* ^2^ = 5.172; *P* = .395).

## 4. Discussion

 Data on prosthetic status and prosthetic need among the institutionalized elderly is very scanty. Therefore, an attempt was made to find out the prosthetic status and prosthetic need of the elderly. 

 The study revealed that 88% of the total population surveyed did not have any prosthesis and none of the subjects had partial dentures. The low proportion of those who had prostheses may be due to the fact that older people underuse dental facilities due to lack of awareness, financial constraints, and reduced mobility; misconceptions regarding adjustments to dentures and lack of interest in aesthetics may also be contributing factors. This finding was in accordance with the findings of Soh et al. [3]. Another finding was that the prosthetic status was slightly better in males than in females. Females usually depend on male members of their families to take them for treatment. Coupled with the lack of access, a lower level of education and lack of employment could be possible reasons for more females being edentulous than males. 

 The need for full prostheses in upper and lower arches was more in comparison to the need for partial dentures, as also found by Slade et al. [4]. The need was slightly more in males than in females, as stated by Singh et al. [5]. In this study, the need for multiunit prostheses was more than the need for single-unit prostheses.

 The authors recommend that higher utilization of care can be achieved by providing on-site dental care instead of referring elderly persons to dental clinics. This may be of immense value in fulfilling the prosthetic need of the elderly, especially the nonambulatory persons.

## 5. Conclusions

 The findings of this study clearly demonstrate a high unmet need for prosthetic care among the institutionalized elderly population surveyed. 

 These results may serve as a baseline reference for the future evaluation of prosthetic status and prosthetic need among the elderly residing in old age homes, and as an eye-opener for oral health service providers and decision makers, thereby sensitizing them to the lacunae existing in the provision of health care for the elderly.

##  Conflict of Interests

This paper has been read and approved by both the authors. There is no conflict of interests with respect to this paper.

## Figures and Tables

**Table 1:** Distribution of study subjects according to their age and gender.

Age groups (in years)	Males *n* (%)	Females *n* (%)	Total *n* (%)
60–65	22 (55%)	18 (45%)	40 (100%)
65–70	10 (33.3%)	20 (66.7%)	30 (100%)
70–75	13 (52%)	12 (48%)	25 (100%)
75–80	11 (61.1%)	7 (38.9%)	18 (100%)
80–85	15 (93.8%)	1 (6.3%)	16 (100%)
85–90	2 (50%)	2 (50%)	4 (100%)

Total	73 (54.9%)	60 (45.1%)	133 (100%)

**Table 2:** Distribution of study subjects according to gender and the prosthetic status of their upper and lower arches.

	Prosthetic status of upper arch			Prosthetic status of lower arch		
	Males *n* (%)	Females *n* (%)	Total	Males *n* (%)	Females *n* (%)	Total
No prosthesis	60 (82.2%)	57 (95%)	117 (88%)	60 (82.2%)	57 (95%)	117 (88%)
Full removable denture	13 (17.8%)	3 (5%)	16 (12%)	13 (17.8%)	3 (5%)	16 (12%)

Total	73 (100%)	60 (100%)	133 (100%)	73 (100%)	60 (100%)	133 (100%)

**Table 3:** Distribution of study subjects according to gender and the prosthetic need of their upper and lower arches.

	Prosthetic status of upper arch			Prosthetic status of lower arch		
	Males *n* (%)	Females *n* (%)	Total	Males *n* (%)	Females *n* (%)	Total
No prosthesis needed	15 (20.5%)	12 (20%)	27 (20.3%)	15 (20.5%)	12 (20%)	27 (20.3%)
Need for one unit prosthesis	0 (0%)	2 (3.3%)	2 (1.5%)	2 (2.7%)	2 (3.3%)	4 (3%)
Need for multiunit prosthesis	13 (17.8%)	18 (30%)	31 (23.3%)	16 (21.9%)	20 (33.3%)	36 (27.1%)
Need for combination of single- or multiunit prosthesis	4 (5.5%)	3 (5%)	7 (5.3%)	4 (5.5%)	3 (5%)	7 (5.3%)
Need for full prosthesis	37 (50.7%)	25 (41.7%)	62 (46.6%)	32 (43.8%)	23 (38.3%)	55 (41.4%)
Not recorded	4 (5.5%)	0 (0%)	4 (3%)	4 (5.5%)	0 (0%)	4 (3%)

Total	73 (100%)	60 (100%)	133 (100%)	73 (100%)	60 (100%)	133 (100%)
